# 循环肿瘤细胞捕获材料的研究进展

**DOI:** 10.3724/SP.J.1123.2021.05020

**Published:** 2021-10-08

**Authors:** Wenjing SUN, Zhenqiang SHI, Guangyan QING

**Affiliations:** 1.中国科学院大连化学物理研究所, 辽宁大连 116023; 2.江南大学药学院, 江苏无锡 214122

循环肿瘤细胞(circulating tumor cells, CTCs)是在肿瘤发展过程中,从肿瘤部位脱落并侵入外周血液中的肿瘤细胞,被认为携带有关肿瘤产生、发展和转移的重要信息,与肿瘤转移密切相关^[1]^。它是肿瘤新转移灶形成的前提,被认为是肿瘤组织转移的“种子”,可看作原发灶肿瘤细胞与转移灶肿瘤细胞之间的中间过渡态。CTCs从实体肿瘤病灶脱落后,借助血液循环系统逃逸并锚定,发展成为新的转移灶,极大地增加了肿瘤患者的死亡风险。据报道,90%的肿瘤患者因肿瘤转移而死亡^[2]^。从患者血液中分离并分析CTCs,有助于监测肿瘤变化程度,诊断治疗效果和预测转移及复发^[3]^。以CTCs为重要生物标志物,设计和开发精准性、特异性CTCs捕获材料^[4]^,以实现对肿瘤发生和发展的实时监测,对肿瘤的早期诊断、术后评估等具有非常深远的临床意义。目前,精准CTCs捕获材料的开发已然成为相关领域的研究热点。本文对CTCs捕获所面临的挑战,以及CTCs捕获材料在近1~2个月内的最新研究进展进行了简要评述。

## 1 CTCs捕获材料的种类

现有的CTCs捕获材料,根据其捕获单元的种类不同,主要可分为基于天然抗体或人工抗体的捕获材料两大类。CTCs表面具有上皮细胞黏附分子(epithelial cell adhesion molecule, EpCAM)、细胞角蛋白(cytokeratin, CK)、癌胚抗原(carcinoembryonic antigen, CEA)和前列腺抗原(prostate specific antigen, PSA)等特异性抗原。基于天然抗体的CTCs捕获材料,是将上述特征标志物的抗体修饰到材料表面,基于抗原-抗体的生物亲和原理,实现对CTCs的特异性捕获。而基于人工抗体的CTCs捕获材料,则是通过模拟抗原-抗体相互作用机理,将能与CTCs表面特征标志物(如唾液酸等)高选择性结合的各种多肽、适配体^[5]^、糖类或其他小分子修饰到材料表面,实现CTCs的选择性捕获。两种材料均存在各自的优势和缺陷,基于天然抗体的捕获材料在特异性方面优势显著。但是,基于人工抗体的捕获材料能够针对目前CTCs捕获面临的诸多问题,更加灵活地进行个性化、多元化功能设计,并且在材料稳定性、制备成本等方面也具有一定优势。

## 2 CTCs捕获材料面临的挑战

CTCs因在血液中的低丰度、异质性,以及捕获过程造成的细胞损伤等原因,导致对CTCs的精准捕获极具挑战^[6]^。如何通过材料设计有针对性地应对上述挑战,是目前CTCs捕获材料的研究重点。

### 2.1 低丰度

血液中含有大量的血细胞,如白细胞(10^9^个/L)、红细胞(10^12^个/L)、血小板(10^12^个/L)等。与这些正常血细胞相比,CTCs在血液中的含量非常低^[7]^。研究表明:每10^5^~10^6^个外周血单核细胞(peripheral blood mononuclear cell, PBMC)中仅有1个CTCs^[8]^。这对捕获材料的灵敏度和特异性提出了非常高的要求。另一方面,血浆蛋白质在材料表面的非选择性吸附也会影响材料对CTCs的捕获性能,这就要求CTCs捕获材料必须具备足够的抗污性能。此外,将来若要实现在血液循环系统中对CTCs的原位实时监测,捕获材料的生物相容性,尤其是血液相容性和细胞相容性,也是不可或缺的性能要素。

近日,柏林自由大学Rainer Haag课题组^[9]^设计了一种基于聚甘油和上皮细胞黏附分子抗体(anti-EpCAM)的纳米结构化涂层(见图1)。该涂层表面修饰的anti-EpCAM抗体可与CTCs表面的EpCAM通过生物亲和作用特异性结合,实现CTCs精准捕获;同时,该涂层的纳米结构模拟细胞外基质(extracellular matrix, ECM)能增加CTCs在材料表面的黏附性,提升捕获效率;更重要的是,涂层上的超支化聚甘油组分增强了材料的抗污染性能,可有效抑制白细胞在材料表面的非选择性黏附,进而提升CTCs的捕获纯度。

**图1:**
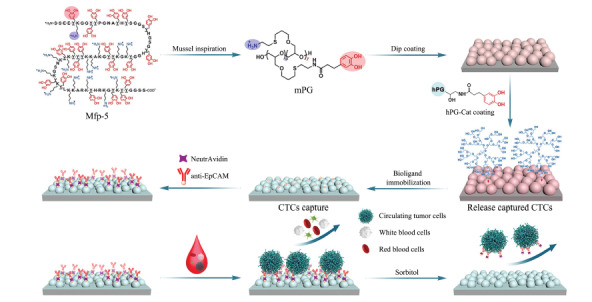
基于聚甘油和anti-EpCAM的纳米结构化多价生物涂层用于CTCs捕获^[9]^

### 2.2 异质性

肿瘤细胞的异质性是指同类肿瘤的瘤间,以及同一肿瘤组织的瘤内,存在形态、功能差异的癌细胞亚群^[10]^。对CTCs而言^[11]^,其异质性主要体现在细胞表面特征标志物种类及数量的动态变化,这将对材料的CTCs捕获效率产生较大影响。

现有基于天然抗体的CTCs捕获材料,主要以CTCs表面的EpCAM为靶标,用相应的抗原实现CTCs捕获。但是,肿瘤转移过程中的上皮-间质转化(epithelial mesenchymal transition, EMT),会使CTCs丢失上皮抗原性,EpCAM表达显著下调^[12,13]^,从而导致以EpCAM为靶标的CTCs捕获效率大大降低,甚至造成假阴性的检测结果。针对这一问题,中国科学院大学胡志远课题组^[14]^以CTCs表面的间质细胞标志物*N*-钙黏蛋白为靶标,从一珠一化合物(one-bead one-compound, OBOC)肽库中筛选出一种能与*N*-钙黏蛋白特异性结合的新型肽,并将其修饰到磁性纳米粒子表面,用于间质CTCs捕获。此类材料是对anti-EpCAM抗体类捕获材料的一个很好补充,有助于克服CTCs因EMT造成的异质性。

另外,西南大学宋尔群课题组^[15]^创新性地利用梯度磁响应性策略,实现了对不同CTCs亚群的特异性捕获与分离(见图2)。他们构筑了一系列不同抗体修饰的磁性纳米粒子探针,通过“鸡尾酒”式多抗体联用,实现了对表面携带不同抗原的多种CTCs亚群的同时捕获。更有意思的是,通过梯度磁响应分离,该纳米探针可将捕获的CTCs,从血液样本中按时间顺序依次分离。CTCs表面抗原的数量,将直接影响与磁性材料结合后CTCs的磁响应性能,利用这一特点,该材料能够很好克服CTCs的异质性,对表面具有不同表达水平的各种标志物的CTCs亚群进行特异性捕获和梯度分离。

**图2:**
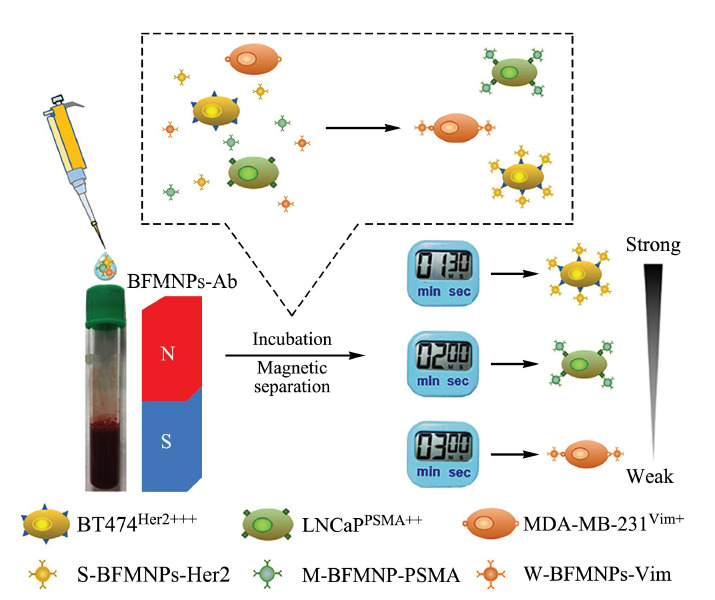
不同抗体修饰的仿生荧光磁性纳米探针(BFMNPs-Ab)联用实现对异质性肿瘤细胞亚群的捕获与磁响应性梯度分离^[15]^

人工抗体材料方面,中国科学技术大学裴仁军课题组^[16]^开发了一种单宁酸功能化的磁性纳米材料,利用单宁酸的多酚单元与癌细胞表面糖萼的相互作用,该材料能够对HeLa、PC-3、T24等7种癌细胞进行广谱性捕获。当与密度梯度离心联用时,该材料能从乳腺癌、肾癌、前列腺癌、肺癌等多种癌症的临床血液样本中捕获CTCs。

### 2.3 细胞损伤

普遍认为,CTCs携带肿瘤部位病变细胞的重要信息。捕获过程引起的细胞损伤会导致CTCs所携带的核酸、蛋白质、多糖等关键信息流失,极大地阻碍了对CTCs的深度分析。此外,现有的CTCs释放策略^[17,18,19]^,如胰酶降解、竞争解离等,往往会引起CTCs应激反应,导致细胞信息丢失、活性降低,从而影响下游细胞培养和分子分析^[20]^。因此,如何实现细胞的无损捕获与释放也是CTCs捕获材料的一大难题。

如图3a所示,厦门大学杨朝勇课题组^[21]^近日报道了一种刺激响应性配体修饰的微流控芯片(stiMulus-responsive ligand-decorated microfluidic chip, MRD-Chip),用于外周血中循环骨髓瘤细胞的有效捕获和无损释放。他们通过二硫键将CD138抗体修饰到微流控芯片上,并通过芯片结构优化,增强了细胞与捕获抗体之间的有效接触,从而实现了对CTCs的高效捕获。更重要的是,基于硫醇与二硫键交换反应,该材料可以实现对被捕获CTCs的精准、无损释放。通过引入二硫苏糖醇(DTT),与CD138抗体相连的二硫键会被还原而发生断键,从而将CD138抗体以及与其特异性结合的CTCs从材料表面精准释放,并且被释放的CTCs的细胞活性保持在90%以上。这一动态二硫键的设计,使材料能在有机小分子还原剂的刺激下,按需触发CTCs的精准、无损释放,避免了胰酶等传统解离试剂对细胞造成的损伤。

**图3:**
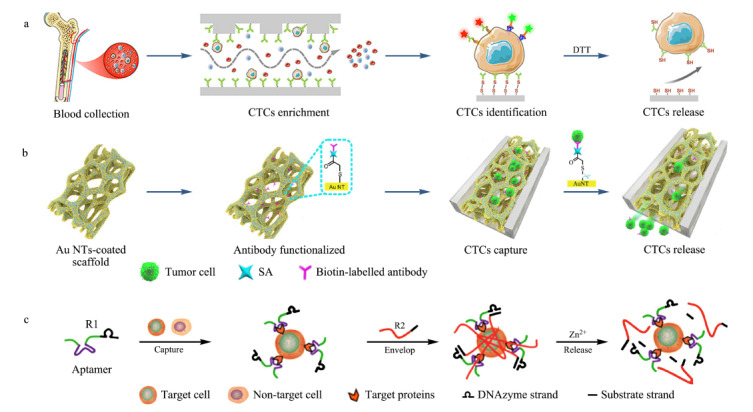
CTCs的刺激响应性无损释放

为了进一步避免刺激响应分子对样品造成的二次污染,武汉大学谢敏课题组^[22]^设计了一种具有电响应性CTCs释放功能的捕获材料(见图3b)。他们利用金属置换法在聚二甲基硅氧烷(PDMS)骨架上原位合成金纳米棒(AuNTs),并通过Au-S键在AuNTs表面共价修饰anti-EpCAM抗体,基于抗体与CTCs表面抗原的生物亲和作用,实现对CTCs的特异性捕获。同时,该材料能够通过电化学刺激触发Au-S键断裂,将与anti-EpCAM抗体特异性结合的CTCs从材料表面释放。这一巧妙设计不仅实现了CTCs的无损释放,而且能在很大程度上提高收集到的CTCs纯度。

基于人工抗体的捕获材料在避免细胞损伤方面同样做出了突出贡献。湖南大学贺建军课题组^[23]^基于脱氧核酶(DNAzyme)水凝胶的溶胶-凝胶-溶胶转化,实现了对CTCs的选择性捕获、原位包封及温和释放(见图3c)。他们利用滚环扩增技术合成了两条单链DNA(R1和R2), R1链包含DNAzyme序列和能特异性识别CTCs表面标志物的适配体序列,R2链包含DNAzyme的互补序列。当R1链与CTCs结合后,R2链的加入可以触发溶胶-凝胶转变,形成DNAzyme水凝胶,实现CTCs的原位捕获与分离。此外,该DNAzyme水凝胶能在锌离子(Zn^2+^)刺激下瓦解,将被捕获的CTCs从凝胶中释放。DNAzyme水凝胶的细胞相容性,以及温和的释放条件,使收集到的CTCs保持了较高的细胞活性。

## 3 总结与展望

作为肿瘤诊疗的有效途径之一,CTCs捕获材料的开发对肿瘤的早期诊断、术后评估等具有非常深远的临床意义。针对目前CTCs捕获所面临的挑战,CTCs捕获材料的重点发展方向主要有:1)提高材料对CTCs捕获的选择性、灵敏度和抗干扰性能;2)扩大对不同CTCs亚群的覆盖度;3)减小捕获过程造成的细胞损伤。由此开发的CTCs精准捕获材料,将有助于实现对肿瘤的原位实时监测,使癌症真正做到“早发现、早诊断、早治疗”。
